# Ventilator-associated pneumonia in ICU patients with severe pneumonia and respiratory failure

**DOI:** 10.1172/JCI172643

**Published:** 2023-06-15

**Authors:** Sarah Jackson

The COVID-19 pandemic resulted in an unprecedented number of patients hospitalized in intensive care units (ICUs) because of severe SARS-CoV-2 infection (1). Respiratory failure in ICU patients, whether due to respiratory infection or other causes, may necessitate mechanical ventilation if oxygen levels cannot be restored with less invasive devices, such as continuous positive airway pressure (CPAP) or bilevel positive airway pressure (BiPAP) ventilators. Although ventilators can be life-saving, their use is associated with some risks, including secondary bacterial infections that cause pneumonia. Prior studies have established that patients with SARS-CoV-2 infection on a mechanical ventilator are more likely to have ventilator-associated pneumonia (VAP) compared with other ICU patients, including those with influenza (2–4). This increased risk of VAP might occur because SARS-CoV-2 infection induces such profound lung injury compared with other infectious insults.

In this issue of the *JCI*, Gao, Markov, Stoeger, and colleagues developed a new approach to assess features that associate with VAP and mortality in a cohort of ICU patients with severe pneumonia and respiratory failure (5). The research team used electronic health records from 585 mechanically ventilated patients hospitalized at Northwestern Memorial Hospital, including 190 patients with COVID-19, 252 patients with bacterial pneumonia, 50 patients with other viral respiratory infections, and 93 control patients who had respiratory failure without pneumonia. The patients with COVID-19 spent more time in the ICU and were mechanically ventilated longer than other patient groups in the study. In order to compare the trajectory of the distinct patient groups in the study with varying lengths of stay in the ICU, the authors developed a machine-learning algorithm called *CarpeDiem* that used 44 different clinical parameters from electronic health records to cluster days with similar patient features. This approach was further validated utilizing electronic health records from the Beth Israel Deaconess Medical Center that are publicly available in the MIMIC-IV database.

This study revealed that unsuccessful treatment of VAP is associated with mortality across all patient groups. While it may be intuitive that unresolving secondary bacterial infection associates with increased mortality risk, the authors provide clear data definitively illustrating the occurrence of subsequent bacterial pneumonia in the different patient groups. Furthermore, their findings suggest that patients with COVID-19 are at higher risk of VAP associated with the prolonged respiratory failure triggered by SARS-CoV-2 infection relative to the other ICU patient groups in this study. An important implication of the work is that better approaches to detect and treat, or prevent, VAP could potentially improve outcomes. Further studies are needed to disentangle the many comorbidities associated with VAP, such as the selection of antibiotics, other drug exposures, and individual risk factors that make patients susceptible. Last, the machine-learning tool developed by the research team may have broader clinical applications in other areas of critical care medicine and could provide an innovative approach to examine other complications and treatments in the ICU.

## Version 1 06/15/2023

Electronic publication
